# Comparative Evaluation of U.S. Brand and Generic Intravenous Sodium Ferric Gluconate Complex in Sucrose Injection: In Vitro Cellular Uptake

**DOI:** 10.3390/nano7120451

**Published:** 2017-12-15

**Authors:** Min Wu, Dajun Sun, Katherine Tyner, Wenlei Jiang, Rodney Rouse

**Affiliations:** 1 Division of Applied Regulatory Science, Office of Clinical Pharmacology, Office of Translational Sciences, Center for Drug Evaluation and Research, U.S. Food and Drug Administration, Silver Spring, MD 20993, USA; min.wu@fda.hhs.gov; 2 Office of Research and Standards, Office of Generic Drugs, Center for Drug Evaluation and Research, U.S. Food and Drug Administration, Silver Spring, MD 20993, USA; dajun.sun@fda.hhs.gov (D.S.); wenlei.jiang@fda.hhs.gov (W.J.); 3 Office of Pharmaceutical Quality, Center for Drug Evaluation and Research, U.S. Food and Drug Administration, Silver Spring, MD 20993, USA; katherine.tyner@fda.hhs.gov

**Keywords:** sodium ferric gluconate complex, parenteral iron, Ferrlecit, generic drugs, cell uptake, bioequivalence

## Abstract

Iron deficiency anemia is a common clinical consequence for people who suffer from chronic kidney disease, especially those requiring dialysis. Intravenous (IV) iron therapy is a widely accepted safe and efficacious treatment for iron deficiency anemia. Numerous IV iron drugs have been approved by U.S. Food and Drug Administration (FDA), including a single generic product, sodium ferric gluconate complex in sucrose. In this study, we compared the cellular iron uptake profiles of the brand (Ferrlecit^®^) and generic sodium ferric gluconate (SFG) products. We used a colorimetric assay to examine the amount of iron uptake by three human macrophage cell lines. This is the first published study to provide a parallel evaluation of the cellular uptake of a brand and a generic IV iron drug in a mononuclear phagocyte system. The results showed no difference in iron uptake across all cell lines, tested doses, and time points. The matching iron uptake profiles of Ferrlecit^®^ and its generic product support the FDA’s present position detailed in the draft guidance on development of SFG complex products that bioequivalence can be based on qualitative (Q1) and quantitative (Q2) formulation sameness, similar physiochemical characterization, and pharmacokinetic bioequivalence studies.

## 1. Introduction

Iron deficiency anemia is a common complication in people who suffer from advanced stages of chronic kidney disease, especially those requiring dialysis. Oral iron therapy is generally recommended for patients with non-dialysis chronic kidney disease (CKD). Intravenous (IV) administration iron supplementation is the preferable treatment for CKD patients undergoing hemodialysis and/or receiving erythropoiesis-stimulating agents (ESA) that increase iron requirements. Although oral iron is a convenient and economical treatment option, the clinical benefits of oral iron drugs have been limited by poor oral bioavailability and drug intolerance due to adverse drug reactions [1]. Research findings have consistently supported the benefits of IV iron over oral iron in treating CKD patients with moderate to severe iron deficiency anemia: rapid repletion of iron stores, significant increase of hemoglobin levels, and a low rate of treatment-related adverse events [2,3,4,5,6].

Patients with hemodialysis are often concurrently prescribed ESA therapy and IV iron that can readily increase iron availability and facilitate erythropoiesis. Currently there are six FDA-approved branded IV parenteral iron therapies: InFed^®^ (low molecular weight iron dextran, Actavis, Parsippany-Troy Hills, NJ, USA), Dexferrum^®^ (high molecular weight iron dextran, Luitpold Pharmaceuticals, Inc., Eagleville, PA, USA), Ferrlecit^®^ (sodium ferric gluconate complex (SFG), Sanofi Aventis US, Bridgewater, NJ, USA), Venofer^®^ (iron sucrose, Luitpold Pharmaceuticals, Inc.), Feraheme™ (ferumoxytol, AMAG Pharmaceuticals, Inc., Waltham, MA, USA), and Injectafer^®^/Ferinject^®^ (ferric carboxymaltose, Luitpold Pharmaceuticals, Inc.). There are more IV iron therapies under investigation. For example, Monofer^®^ (iron isomaltoside 1000, Pharmacosmos, Holbæk, Denmark) is in a Phase III clinical trial at this time [7,8]. All of the above products are iron colloids with an iron core surrounded by different carbohydrate coatings [9,10,11]. U.S. Food and Drug Administration (FDA) guidance recommends that the proposed generic versions of IV iron products have the same carbohydrate coating as the brand product they are referencing. Differences in the carbohydrate coatings can change the safety profiles of the products and lead to adverse drug events [12,13,14]. For example, anaphylactic or anaphylactoid reactions and death caused by severe immunologic responses to the dextran carbohydrate shell have led to an FDA change in the labeling of Dexferrum^®^ [12,15,16]. In the case of iron dextran, low molecular weight iron dextran tends to produce fewer adverse events than high molecular weight iron dextran. Ferrlecit^®^ and Venofer^®^ have demonstrated improved patient compliance and reduced adverse drug events [13,17,18]. The enhanced efficacy is proposed to be a result of increased iron donation to transferrin [19] and a rapid transferrin saturation rate [20]. Although iron gluconate and iron sucrose generally have similar safety outcomes, it has been observed that iron gluconate increased labile iron and lipid oxidation [21], while iron sucrose presented a higher risk of infection-related consequences in patients on hemodialysis [22]. Thus, it is important to assess the pharmaceutical equivalence of generic and brand SFG using a weight-of-evidence approach, characterizing the products based on the recommended studies in product-specific guidance [23,24,25,26,27].

Parenterally administrated iron colloids are internalized by the phagocytic cells of the mononuclear phagocyte system (MPS). To maintain iron homeostasis, iron is either stored in ferritin/hemosiderin or transported by transferrin to its ultimate site of action such as the bone marrow for erythropoiesis. Iron release from the colloidal suspension and its subsequent distribution are size- and surface-dependent, with differences in core size and carbohydrate chemistry determining pharmacologic and bioactivity differences such as clearance rate, iron release rate, maximum tolerated dose, and rate of infusion [28,29]. Compared to iron dextran, the binding between the components is looser in iron sucrose and iron gluconate, which facilitates iron dissociation. This weak bond, however, increases the release of labile plasma iron, causing oxidative stress and potential tissue injury [19,30,31,32]. Currently, only one generic iron colloidal IV product, which was approved in 2011, is on the U.S. market (a generic SFG, Watson Pharma Inc., Corona, CA, USA). The FDA published a product-specific guidance for SFG IV infusion products [24] and the European Medicines Agency (EMA) published a reflection paper [33]. In FDA’s draft product-specific guidance, generic applicants need to demonstrate bioequivalence between generic SFG with its reference-listed product (RLD) via qualitative (Q1) and quantitative (Q2) formulation sameness, similar physicochemical characterization (e.g., particle size distribution, iron core characterization, composition of carbohydrate shell, particle morphology, and labile iron determination) and in vivo pharmacokinetic bioequivalence studies [24]. In addition to the above recommended studies, EMA further requests non-clinical studies of nanoparticle iron uptake and distribution in plasma, macrophages, pharmacological/toxicological target tissues in animal and in vitro cellular model [33]. To compare the different approaches to equivalence between the U.S. FDA and EMA, we conducted in vitro cellular uptake studies to compare iron uptake in human macrophage cells treated with two lots of Ferrlecit^®^ and one lot of the generic SFG. The information obtained through this study supports the quality of U.S.-approved generic iron complex products and FDA’s review standards for SFG complex drug products.

## 2. Results

Commercially available lots of Ferrlecit^®^ and generic SFG compliant with FDA-approved specifications were purchased from a retail pharmacy. These products were fully characterized and the generic SFG has similar particle size distribution, iron core, composition of carbohydrate shell, and other physicochemical properties as the reference drug Ferrlecit^®^ [34]. In this study, the iron uptake comparison between Ferrlecit^®^ and the generic SFG was performed in three different human macrophage cell lines: THP-1, HL60, and U937. All three macrophage cell lines have been extensively used in research to study nanoparticle uptake [35,36,37,38,39]. For instance, the THP-1 cell line was used to study the iron uptake by comparing Venofer^®^ and its generic iron sucrose [36].

**Figure 1:**
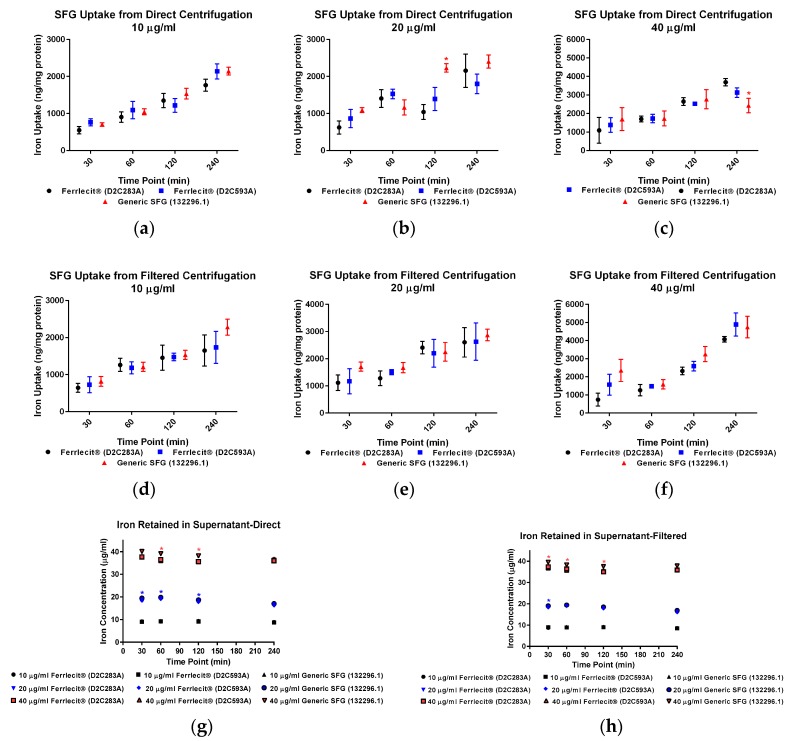
Comparison of iron uptake in THP-1 cells. Data are presented as mean ± SD (*n* = 3, three independent experiments with four replicates for each experimental condition). A *p*-value < 0.05 was considered statistically significant, marked with *. (**a**) Iron uptake in THP-1 cells treated with 10 µg/mL drugs was measured from direct centrifugation harvest; (**b**) iron uptake in THP-1 cells treated with 20 µg/mL drugs was measured from direct centrifugation harvest; (**c**) iron uptake in THP-1 cells treated with 40 µg/mL drugs was measured from direct centrifugation harvest; (**d**) iron uptake in THP-1 cells treated with 10 µg/mL drugs was measured from filtered centrifugation harvest; (**e**) iron uptake in THP-1 cells treated with 20 µg/mL drugs was measured from filtered centrifugation harvest; (**f**) iron uptake in THP-1 cells treated with 40 µg/mL drugs was measured from filtered centrifugation harvest; (**g**) iron retained in cell culture supernatant was measured from direct centrifugation harvest; (**h**) iron retained in cell culture supernatant was measured from filtered centrifugation harvest.

**Figure 2:**
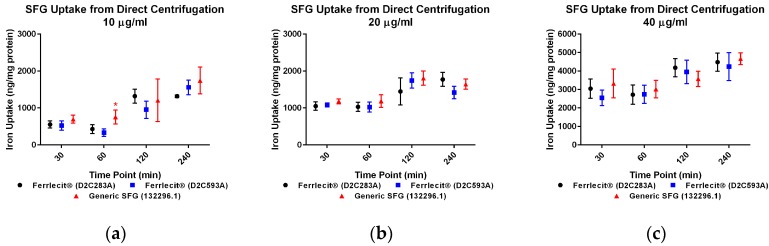

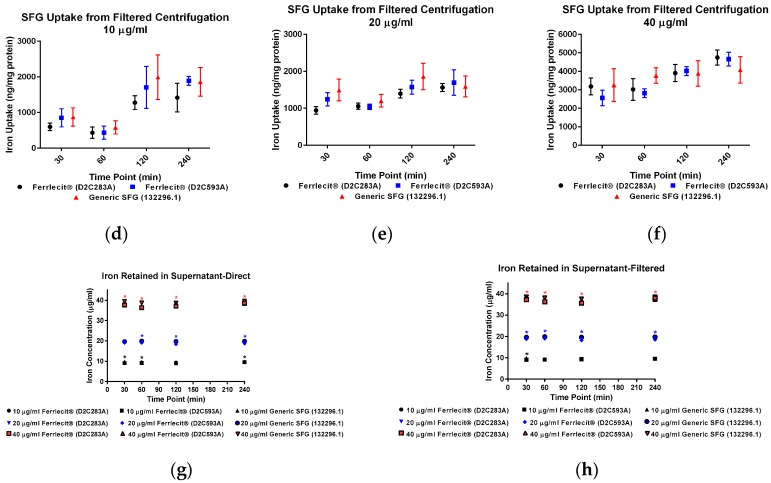
Comparison of iron uptake in HL-60 cells. Data are presented as mean ± SD (*n* = 3, three independent experiments with four replicates for each experimental condition). A *p*-value < 0.05 was considered statistically significant, marked with *. (**a**) Iron uptake in HL-60 cells treated with 10 µg/mL drugs was measured from direct centrifugation harvest; (**b**) iron uptake in HL-60 cells treated with 20 µg/mL drugs was measured from direct centrifugation harvest; (**c**) iron uptake in HL-60 cells treated with 40 µg/mL drugs was measured from direct centrifugation harvest; (**d**) iron uptake in HL-60 cells treated with 10 µg/mL drugs was measured from filtered centrifugation harvest; (**e**) iron uptake in HL-60 cells treated with 20 µg/mL drugs was measured from filtered centrifugation harvest; (**f**) iron uptake in HL-60 cells treated with 40 µg/mL drugs was measured from filtered centrifugation harvest; (**g**) iron retained in cell culture supernatant was measured from direct centrifugation harvest; (**h**) iron retained in cell culture supernatant was measured from filtered centrifugation harvest.

**Figure 3:**
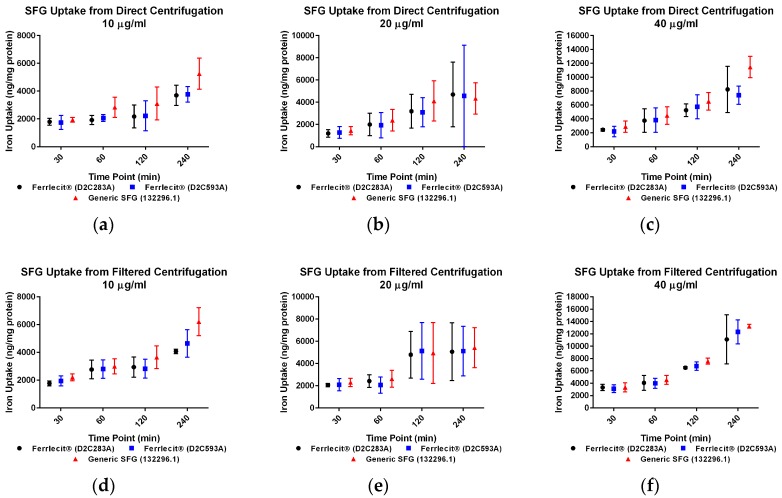

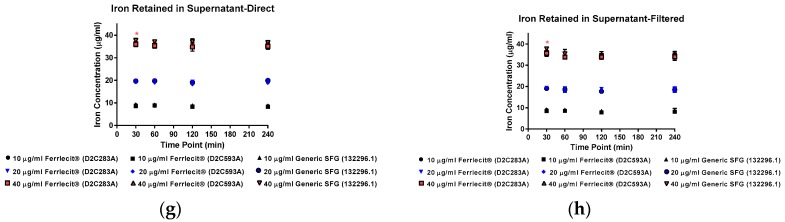
Comparison of iron uptake in U937 cells. Data are presented as mean ± SD (*n* = 3, three independent experiments with four replicates for each experimental condition). A *p*-value < 0.05 was considered statistically significant, marked with *. (**a**) Iron uptake in U937 cells treated with 10 µg/mL drugs was measured from direct centrifugation harvest; (**b**) iron uptake in U937 cells treated with 20 µg/mL drugs was measured from direct centrifugation harvest; (**c**) iron uptake in U937 cells treated with 40 µg/mL drugs was measured from direct centrifugation harvest; (**d**) iron uptake in U937 cells treated with 10 µg/mL drugs was measured from filtered centrifugation harvest; (**e**) iron uptake in U937 cells treated with 20 µg/mL drugs was measured from filtered centrifugation harvest; (**f**) iron uptake in U937 cells treated with 40 µg/mL drugs was measured from filtered centrifugation harvest; (**g**) iron retained in cell culture supernatant was measured from direct centrifugation harvest; (**h**) iron retained in cell culture supernatant was measured from filtered centrifugation harvest.

We collected both cell pellets and cell culture supernatant to directly and indirectly determine the cellular iron uptake with and without centrifuge tube filters. The adapted colorimetric assay was able to measure iron both as an element and in a complex. Based on the cell pellet sample results, in general, the iron uptake profile exhibits a consistent pattern across the tested cell lines and doses with a time-dependent increase. Figure 1a–f showed that the amount of iron taken up by THP-1 cells increased with a longer incubation time. Similar results were obtained in HL-60 cells (Figure 2a–f) and U937 cells (Figure 3a–f), indicating that iron uptake was positively correlated with incubation time.

When measuring the iron content in cell pellets from direct collection (no centrifuge tube filters) at the low dose (10 µg/mL) in THP-1 cells (Figure 1a) and U937 cells (Figure 3a), there was no statistically significant difference of iron uptake between the assayed Ferrlecit^®^ and the generic SFG at each time point. A similar iron uptake profile between the two drugs was also observed in HL-60 cells at most time points (Figure 2a). When the dose increased close to the Cmax in humans (20 µg/mL), the iron uptake between the brand and the generic SFG in all three cell lines consistently showed no statistical difference at the tested time points (Figure 1b). Once the dose reached a value double the Cmax in humans (40 µg/mL), the iron uptake was similar between the brand and the generic SFG at all time points except for different iron uptake after 240 min incubation in THP-1 cells (Figure 1c).

When measuring the iron content in cell pellets collected through the centrifuge tube filters, there was no statistical difference in iron uptake between the three cell lines across all the time points (Figure 1d–f, Figure 2d–f and Figure 3d–f). Iron uptake was consistent between cells collected with or without filtration and exhibited a positive correlation with time. Since the incubation time is 4 h or less, it is not surprising that the majority of iron was retained in the cell culture supernatant in each cell line (Figure 1g,h, Figure 2g,h and Figure 3g,h). As expected, the iron in cell culture supernatant decreased with increasing incubation time in all three cell lines with all SFG lots. In THP-1 cells, no statistical difference between brand and generic drugs were observed in cell culture supernatant at 10 µg/mL (Figure 1g,h), In HL-60 cells, consistent differences between products existed at all doses (Figure 2g,h). In U937 cells, a consistent and similar iron decrease in cell culture supernatant was noted in both brand and generic products at all time points and doses except the high dose (40 µg/mL) after 30 min incubation (Figure 3g,h).

## 3. Discussion

We have previously compared the physicochemical characterization of Ferrlecit^®^ and the generic SFG and found no difference in elemental analysis, thermal properties, particle size, or zeta potential molecular weight determined by gel permeation chromatography (GPC), but slight differences in the sedimentation coefficient determined by analytical ultracentrifugation (AUC) and molecular weight by asymmetric field flow fractionation–multi-angle light scattering (AFFF–MALS) [34].

In addition to a biodistribution study in rats [40], we conducted in vitro cellular uptake studies to compare the iron uptake of the brand IV iron drug and its generic version in MPS. Macrophages are among the first players responding to nanoparticle exposure. They are also the primary cells involved in nanoparticle uptake, processing, and signal transduction. Multiple groups have studied nanoparticle uptake in in vitro systems. Agglomeration, sedimentation, and diffusion are believed to impose effects on real nanoparticle uptake. Cho et al. considered sedimentation effects when examining large and heavy nanoparticles [41], and invented an inverted system showing that sedimentation increased the real gold nanoparticle uptake. In another study, Bancos and Tyner compared gold nanoparticle uptake in RAW264.7 cells over 72 h under static plate-based and insert-based culture conditions [42]. Their results showed consistently greater intake in the traditional culture setup over time. Here we used human non-adherent macrophage cells to avoid the impact of sedimentation, as noted in previous studies [41,42]. For this reason, cells will likely only incorporate iron from the surrounding media. However, in our assay setup, iron nanoparticles may form a pellet along with the cell pellets during centrifugation. Thus, agglomerations co-precipitated during cell collection could create an artifact. Therefore, centrifuge tube filters were utilized to diminish the impact of agglomeration. Since the pore size for the filter was 0.45 µm, agglomerations larger than the pore could be blocked with cell pellets accumulating on the filter membrane. Conversely, the cell pellets recovered from the centrifuge tube filter membrane could be affected by the properties and quality of the membrane and the retention of larger agglomerates. In addition, there was some adhesion and not all cells could be recovered from the filter membrane. The iron uptake values of Ferrlecit^®^ and the generic SFG in cell pellets obtained from direct centrifuge and via filter centrifuge tubes were largely consistent using the same experimental conditions. Minor differences and decrement were observed at three experimental conditions (Figure 1b,c and Figure 2a) but lacked consistency and were interpreted as random occurrences. In all three tested cell lines, the amount of cellular iron uptake exhibited similar time- and dose-dependent increases.

The three macrophage cell lines applied in this study differ in their origin and maturation stage. U937 cells are of tissue origin, thus at a more mature stage, whereas THP-1 and HL-60 cells originate from a leukemic patient and are less mature. They are grown in suspension conditions and can be stimulated to a macrophage-like phenotype by phorbol 12-myristate 13-acetate (PMA). A recent study examined whether different RAW264.7 macrophage phenotypes influenced silica nanoparticle uptake [43]. M1 RAW264.7 phenotype had higher silica nanoparticle uptake, indicating the significant involvement of in vivo uptake of this phenotype. Interestingly, iron uptake profile of both drugs is similar in THP-1 cells and HL-60 cells, while U937 cells exhibited higher iron incorporation values, especially with longer incubation time at higher doses. For instance, when treated with 20 µg/mL or 40 µg/mL for 240 min, U937 cells incorporated triple the amount of iron compared to THP-1 cells and HL-60 cells. Therefore, we might predict that more mature macrophage cells could demonstrate greater SFG complex uptake. Moreover, Lunov et al. showed that PMA-differentiated THP-1 cells could change their nanoparticle uptake profiles and mechanisms compared to undifferentiated THP-1 cells [38]. Thus, future activated macrophage cells or more macrophage-like cells studies could provide additional valuable information. The results also highlight the importance of choosing appropriate in vitro cell system to address the desired endpoint.

When nanoparticles are introduced into the in vitro biological system, they can rapidly interact with the serum proteins, generating a dynamic protein corona layer. The protein corona alters nanoparticle physiochemical properties, affects its interaction with cells, and defines the biological properties of nanoparticles [44,45]. It is the nanoparticle-protein corona that affects cellular uptake and nanoparticle internalization in living cell systems. On the other hand, the nature of the nanoparticles also determines the composition of proteins in the corona. Even for a specific nanoparticle material type, the size of the nanoparticle and its surface modifications are able to considerably change the nature of the biologically active proteins in the corona and thereby change the cellular uptake [46]. Different from THP-1 and U937 cells cultured in RPMI 1640 medium supplemented with 10% fetal bovine serum (FBS), HL-60 cells were incubated in IMDM with 20% FBS. Although albumin is the most abundant protein in the FBS and is also the main corona component after incubation in cell culture media, the stability of the protein corona requires the presence of other adsorbed proteins on the particle’s surface [47]. Cheng et al. presented different Au nanoparticle uptake profiles in RAW264.7 cells in the absence and presence of serum [48]. More FBS content, different cell culture medium components, and distinct cell membrane properties account for different formations of the protein corona, thus causing distinct cellular iron uptake profile in HL-60 cells. Understanding the composition and properties of protein corona can help explain cell type-specific nanoparticle uptake, facilitating the development of targeted nanomaterial delivery. Besides the direct measurement of iron uptake in cells, we also measured the amount of iron in the cell culture supernatant. In fact, the applied iron uptake assay measured not only the iron nanoparticles retained in the cell culture media, but also the iron content from FBS and medium. Compared to THP-1 and U937 cells, the consistent variation in iron retained in the HL-60 culture medium may result from protein corona layer differences (Figure 1g,h, Figure 2g,h and Figure 3g,h). The variance detected in iron content in collected cell culture media may indicate a slight dissimilarity between the brand and the generic SFG. However, whether this difference impacts the cellular iron uptake is questionable, as similarity of iron uptake profile was detected in all three macrophage cell lines. The development of appropriate in vitro cell systems to accurately predict the in vivo functions of nanoparticles is challenging because of the dynamic changes of their intrinsic physicochemical properties upon dispersion in biological fluids. There is still an interpretation gap between the in vitro tests and the in vivo system.

There are currently six-FDA approved brand parenteral iron drugs and only one generic SFG complex in sucrose injection on the U.S. market. The assurance of bioavailability, stability, and potential toxicity in vitro is important for IV iron colloidal products. The sole generic SFG complex in sucrose injection product on the U.S. market was approved by the FDA in March 2011 via a bioequivalence approach of qualitative (Q1) and quantitative (Q2) formulation sameness, in vivo pharmacokinetic studies, and similar physicochemical characterization between the brand and generic formulations. In the same year, the European Medicines Agency (EMA) published a reflection paper on the non-binding data requirements of follow-on nanoparticle iron medicinal product applications, recommending the studies requested by the FDA with additional non-clinical studies (e.g., characterization of RES uptake, biodistribution in animal models) [33]. Coupled with the iron biodistribution study in rats [40], the current in vitro cellular uptake studies have confirmed that the FDA’s currently recommended bioequivalence studies for SFG complex drug products are sufficiently sensitive to capture potential differences between brand and generic formulations in the absence of other non-clinical studies recommended by the EMA.

## 4. Materials and Methods 

### 4.1. Chemicals

FBS, Dulbecco’s phosphate buffered saline (DPBS), and Penicillin/Streptomycin (p/s) were purchased from American Type Culture Collection (ATCC, Manassas, VA, USA). RPMI 1640 and IMDM culture media were purchased from Life Technologies (Grand Island, NY, USA). The 100 mM HEPES and 100 mM sodium pyruvate solutions were purchased from Fisher Scientific (Pittsburgh, PA, USA). NaOH, 2-mercaptoethanol, KMnO_4_, ferrozine, neocuproine, ammonium acetate, ascorbic acid, and Corning^®^ Costar^®^ Spin-X^®^ centrifuge tube filters were purchased from Sigma-Aldrich (St. Louis, MO, USA). The 1000 µg/mL Iron (Fe) pure single-element standard (2% HNO_3_) was obtained from PerkinElmer (Waltham, MA, USA). The sodium ferric gluconate complex in sucrose injection used in this study was branded Ferrlecit^®^ (Lot #D2C283A and Lot #D2C593A, Sanofi U.S. LLC, Bridgewater, NJ, USA) and generic SFG (Lot #132296.1, Watson Pharma Inc., Parsippany, NJ, USA). Detailed physicochemical characterizations of the Ferrlecit and generic SFG lots were reported by Sun et al. [34]. The concentrations were 10, 20, and 40 µg/mL, as determined by the post-approval pilot study (PER9801).

### 4.2. Human Mononuclear Phagocyte Cell Lines

Three human monocytic cell lines of THP-1, HL-60, and U937 cells were purchased from ATCC. THP-1 cells are immortalized monocytes that can be cultured in vitro up to passage 25 without changes of cell sensitivity and activity. HL-60 was first isolated from a patient with acute myeloid leukemia, while U937 is a pro-monocytic, human myeloid leukemia cell line. Upon arrival, cells were propagated and aliquots were stored in liquid nitrogen. THP-1 cells were cultured in RPMI 1640 medium supplemented with 10% FBS, 1 mM sodium pyruvate, 10 mM HEPES, 0.05 mM 2-mercaptoethanol, and 1% p/s. THP-1 cells were passaged 3–20 times, after which a new frozen aliquot was used. HL-60 cells were cultured in IMDM medium supplemented with 20% FBS and 1% p/s. HL-60 cells were passaged 3–10 times, after which a new frozen aliquot was used. U937 cells were cultured in RPMI 1640 medium supplemented with 10% FBS, 1 mM sodium pyruvate, 10 mM HEPES, and 1% p/s. U937 cells were passaged 3–10 times, after which a new frozen aliquot was used. All three cell lines were incubated under the condition of 5% CO_2_/37 °C.

### 4.3. Drug Dose Selection and Cellular Uptake Study

According to the pharmacokinetic study with Ferrlecit^®^, the peak drug levels (Cmax) were significantly affected by both the dosage and rate of administration. In the study, the Cmax varied from 9.4 to 20.6 mg/L [49]. The terminal elimination half-life for the drug bound iron only depended on the dosage. It ranged from 0.85 to 1.45 h. Therefore, three doses (10, 20, and 40 µg/mL) at four time points (30 min; 1, 2, and 4 h) were established for the cellular uptake protocol. Prior to starting the project, a small pilot study was constructed to test whether drugs were toxic to cells. The cell viability was checked before and after incubating at 40 µg/mL for 4 h using trypan blue. We did not observe the cell viability change due to drug toxicity (data not shown). Before seeding cells to measure iron uptake, cell viability was determined by trypan blue. Cells with more than 90% viability were seeded in 12-well plates at a density of 7~9 × 10^5^ cells/mL, then SFG treatments were applied at a final concentration of 10, 20, or 40 µg/mL. Cells were incubated with drugs at 5% CO_2_/37°C for less than 10 s, 30 min, or 1, 2, or 4 h. After that, half the volume of cells and cell culture supernatant from the same well were separated by centrifugation in regular Eppendorf tubes and the other half were separated via Corning^®^ Costar^®^ Spin-X^®^ centrifuge tube filters (0.45 µm). After centrifugation, cell culture supernatant was collected. Cell pellets were recovered from the filter membrane if using centrifuge tube filters. Cell pellets were washed twice with ice-cold PBS before storage at −20 °C for future iron content analysis. For each treatment, three independent experiments were performed, with four replicates each time. The applied drug concentrations caused no toxicity in in vitro culturing conditions.

### 4.4. Iron Uptake Assay

The assay was described by Riemer et al. with some modifications [50]. Briefly, the cells were lysed with 500 µL of 50 mM NaOH for 2 h on a shaker. After that, 100-µL aliquots of cell lysate (or 10 µL collected supernatant diluted with 90 µL 50 mM NaOH) were placed in Eppendorf tubes and mixed with 100 µL of 10 mM HCl, and 100 μL of the iron-releasing reagent (a freshly mixed solution of equal volumes of 1 M HCl and 4.5% (w/v) KMnO_4_ in H_2_O). These mixtures were incubated for 2 h at 60 °C within a fume hood. After the mixtures cooled to room temperature, 30 μL of the iron-detection reagent (6.5 mM ferrozine, 6.5 mM neocuproine, 2.5 M ammonium acetate, and 1 M ascorbic acid dissolved in water) was added to each tube. After 30 min, 300 μL of the solution in each tube were transferred into a well of a 96-well plate and the absorbance was measured at 550 nm on a microplate reader. The relative iron concentration was calculated according to the iron standard.

Protein concentration for each sample was measured using a Bio-Rad protein assay kit II (Hercules, CA, USA), according to the manufacturer’s protocol. Briefly, 10 µL of lysates were added to a well of a 96-well plate. The dye concentrate 5× was diluted with H_2_O to working concentration and passed through a 0.22-µm filter system. Two hundred microliters of filtered dye were added to each 10-µL sample and incubated for 5 min before reading the absorbance at 595 nm. The relative protein concentration was calculated according to the protein standard. The final cellular uptake iron was normalized to the amount of protein in the same sample.

### 4.5. Calculation and Statistics

The iron uptake values obtained for cells incubated with three different dosages (10, 20, and 40 µg/mL) for less than 10 s served as the iron baseline for each dosage. The iron uptake values presented in the figures were corrected by subtracting the baseline values from the original calculated values. All values are presented as mean ± SD. Statistical comparison among the values obtained for each group were made by grouped *t*-test or one-way analysis of variance (ANOVA) in GraphPad Prism 6.0 (La Jolla, CA, USA). A *p*-value <0.05 was considered statistically significant, marked with *.

## 5. Conclusions

Our previous report demonstrated comparable quality attributes between two therapeutically equivalent SFG products at formulation, nanoparticle, and polymer levels, except for slight differences observed in AUC and AFFF-MALS analyses. In the current in vitro cellular uptake studies, there is no noticeable difference in the cellular iron uptake in three human macrophage cell lines between brand and generic SFG complex products, despite small differences in iron retention in the supernatant, which may or may not reflect subtle differences between Ferrlecit^®^ and the generic SFG. Coupled with the biodistribution study in rats, the non-clinical results support the same level of iron uptake and biodistribution of Ferrlecit^®^ and the generic SFG in the inactivated macrophage cell lines and rat tissues, respectively.
